# Experimental Validation of Hydrogen Affinity as a Design Criterion for Alloys

**DOI:** 10.3390/ma17246106

**Published:** 2024-12-13

**Authors:** Katarína Nigutová, Lenka Oroszová, Zuzana Molčanová, Dávid Csík, Katarína Gáborová, Jens Möllmer, Marcus Lange, Karel Saksl

**Affiliations:** 1 Institute of Materials Research, Slovak Academy of Sciences, Watsonova 47, 040 01 Košice, Slovakia; nigutova@saske.sk (K.N.);; 2 Faculty of Materials, Metallurgy and Recycling, Technical University of Košice, Letná 9, 042 00 Kosice, Slovakia; 3 Institut für Nichtklassische Chemie e. V., Permoserstraße 15, 04318 Leipzig, Germany

**Keywords:** metal hydrides, high-entropy alloys, hydrogen sorption, hydrogen affinity

## Abstract

This study introduces an innovative approach to alloy design by experimentally validating the semi-empirical concept of Griessen and Driessen, which predicts the hydrogen affinity of solid solutions. The work focuses on designing and synthesizing four equiatomic high-entropy alloys (HEAs) with compositions tailored to exhibit highly endothermic enthalpies of solution and formation, resulting in resistance to hydrogen absorption. Unlike conventional studies that prioritize hydrogen storage capacity, this research uniquely targets alloys optimized for minimal hydrogen interaction, addressing critical needs in hydrogen storage and transportation technologies prone to hydrogen embrittlement. Experimental results confirm the negligible hydrogen absorption of these alloys, with a maximum of 0.23 wt.% (H/M = 0.13) at 2 MPa and 175 °C. This study not only demonstrates the applicability of a theoretical model to guide alloy design but also highlights the potential of these materials for low-pressure hydrogen storage systems, where mechanical integrity and resistance to hydrogen degradation are paramount. The findings bridge the gap between theoretical predictions and practical applications, offering a novel perspective on alloy development for hydrogen-related technologies.

## 1. Introduction

The transition to a hydrogen economy offers a promising pathway to decarbonize the energy sector, which is currently reliant on fossil fuels. Effective hydrogen storage is pivotal to this transition, requiring safe, efficient, and economically viable solutions. Solid-state storage, with its potential for higher hydrogen density compared to liquid or gaseous methods, has emerged as a promising avenue. The selection of appropriate materials for safe and efficient hydrogen storage is crucial. Hydrogen storage systems for a hydrogen-based economy must be efficient, lightweight, highly safe, affordable, and small. Consequently, metal hydrides have been extensively studied as solid-state hydrogen storage materials [1,2]. Their main benefits include the high volumetric density of hydrogen and the ability to absorb and release hydrogen with small pressure changes. Advances in metal hydride technology demonstrate that these materials offer reliable safety for hydrogen storage, reversibility in hydrogen volumetric energy densities, efficient hydrogenation and dehydrogenation processes, low energy requirements, and the use of low-pressure equipment, making them suitable for both mobile and stationary applications [3,4,5,6,7]. Hydrogenation, the process of adding hydrogen molecules to a material, plays a critical role in various sectors, including catalysis, energy storage, and chemical production. In the context of energy storage, hydrogenation can enhance the performance of high-entropy materials by improving their ability to absorb and release hydrogen. Hydrogen storage systems are crucial to advancing sustainable energy solutions, and by optimizing the interaction between hydrogen and high-entropy materials, researchers aim to develop storage systems that are efficient, safe, and effective.

Among the alloys designed for hydrogen absorption storage, so-called high-entropy alloys (HEAs) are currently the most investigated [8,9,10,11,12]. They are highly attractive for industrial applications due to their atomic randomness and superior chemical and physical properties compared to conventional alloys. They possess several key characteristics, including the high-entropy effect, lattice distortion, slow diffusion, and the cocktail effect, which contribute to their versatility and distinctiveness among advanced materials. The selection of elements and their relative proportions play a critical role in defining the properties and performance of HEAs. With their vast range of possible compositions, HEAs can be precisely tailored to achieve desired properties such as phase formation, stability, and optimized processing conditions. Furthermore, HEAs offer significant advantages over conventional alloys, including improved operational efficiency, enhanced productivity, and greater sustainability. They are capable of safely storing more than 3 wt.% of hydrogen or, expressed alternatively, up to 2.5 hydrogen atoms chemically bonded to one metal atom (H/M parameter). HEAs are multicomponent alloys formed of four or more elements. In their strictest definition, these elements are present in equal atomic proportions, while in a broader sense, they contribute between 5% and 35% to the alloy’s total atomic composition. These alloys typically adopt standard lattice structures such as hexagonal close-packed (HCP), body-centered cubic (BCC), or face-centered cubic (FCC) [13,14,15].

The design of these alloys typically focuses on achieving a single-phase high-entropy alloy (HEA) with a BCC structure, which offers numerous interstitial sites (up to 18) for hydrogen storage. A key aspect of this design is the ability to predict the alloy’s affinity for forming interstitial hydrides. While this approach has been less common, a recent review [16] highlighted the semi-empirical concept developed by Griessen and Driessen [17] for determining the hydrogen affinity of solid solutions. The authors found a strong correlation between the enthalpy of solution at infinite dilution (Δ*H_∞_*) and the enthalpy of formation of the concentrated hydride (Δ*H_f_*) and the amount of hydrogen stored in 55 previously studied HEAs.

The objective of this study was to experimentally validate the semi-empirical model to predict the hydrogen absorption capacity of alloys. Four alloys with highly endothermic formation enthalpies were synthesized. According to the model, these alloys were not expected to form hydrides. To test this hypothesis, the alloys were subjected to hydrogen absorption experiments under isobaric conditions (2 MPa H₂) at temperatures up to 250 °C.

## 2. Materials and Methods

### 2.1. Materials Design

First, we used established empirical rules to predict whether our investigated alloys would fall into the solid-solution (high-entropy) region. In the empirical approach, factors representing discrepancies in atomic sizes (*δ*), the concentration of valence electrons (*VEC*), the enthalpy of mixing (Δ*H_mix_*), and a parameter *Ω*, which relates the enthalpy of mixing, entropy of mixing (Δ*S_mix_*), and melting temperature (*T_m_*), are calculated using the following formula [18,19]:

(1) $$\delta =\sqrt{\sum c_{i}{\left(1-\frac{r_{i}}{\bar{r}}\right)}^{2}\;}\times 100$$

(2) $$VEC=\sum c_{i}{\;VEC}_{i}$$

(3) $$∆H_{mix}=\sum_{i<j}4{\;H}_{ij}{\;c}_{i}{\;c}_{j}$$

(4) $$\Omega =\frac{T_{m}∆S_{mix}}{\left|∆H_{mix}\right|}$$

where

(5) $$T_{m}=\sum_{i=1}^{n}c_{i}\;{\left(T_{m}\right)}_{i}$$

and

(6) $$∆S_{mix}=-R\sum c_{i}\ln c_{i}$$

In the equations, *r_i_* and *VEC_i_* stand for the atomic radius and valence electron concentration of element *i*, respectively; $\bar{r}=\sum c_{i}r_{i}$ is the average of atomic radius; *c_i_* and *c_j_* are the atom fractions of elements *i* and *j*; *H_ij_* is the enthalpy of mixing of elements *i* and *j* at the equimolar concentration in regular binary solutions; (*T_m_*)*_i_* is the melting temperature of element *i*; and *R* is the universal gas constant [18,20].

**Figure 1:**
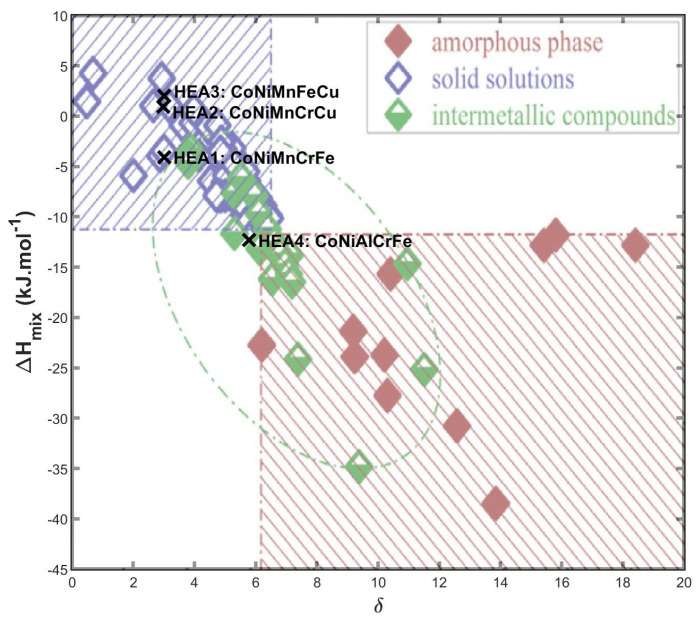
Prediction map of the alloys: the dash-dotted regions indicate areas representing the formation of solid solutions, intermetallic compounds, and amorphous phases (graph adapted from [18]), together with the plotted parameters of the investigated alloys.

Thermodynamic parameters of the investigated alloys are shown in Table 1. A graphical representation of the relationship between the *δ* − Δ*H_mix_* parameters is shown in Figure 1. The region of stability of saturated solid solutions is defined by the parameter of the difference in the size of atoms *δ* < 6.6 % and Δ*H_mix_* falling in the range −11.6 to 3.2 kJ∙mol^−1^ [18,21]. Also, if *Ω* > 1, the Gibbs free energy is designated primarily by the mixing entropy Δ*S_mix_*, which stabilizes the solid solution. The comparison of the thermodynamic parameters listed in Table 1 and Figure 1 shows that our prepared materials can be categorized as high-entropy alloys (Δ*H_mix_* > 1.5*R*), forming a single-phase system. 

#### Hydrogen Affinity

Hydrogen affinity refers to an alloy’s ability to form an interstitial hydride. This affinity can be qualitatively approximated using Griessen and Driessen’s model, which considers the enthalpy of solution at infinite dilution (Δ*H_∞_*) and the enthalpy of formation of the concentrated hydride (Δ*H_f_*) [16,17]. For the highest theoretical hydrogen affinity, both the enthalpy of solution at infinite dilution (Δ*H_∞_*) and the enthalpy of formation of the concentrated hydride (Δ*H_f_*) are calculated as follows:

(7) $$∆H_{\infty }=\sum_{i=1}{\;{\;c}_{i}H}_{\infty i}$$

where *c_i_* is the stoichiometry of the *i*th element and Δ*H_∞i_* is the enthalpy of solution at infinite dilution for the pure metal *i*, and

(8) $$∆H_{f}=\sum_{i=1}{\;{\;c}_{i}H}_{fi}$$

where *c_i_* is the stoichiometry of the *i*th element and Δ*H_fi_* is the enthalpy of formation of the concentrated hydride for the pure metal *i*.

**Figure 2:**
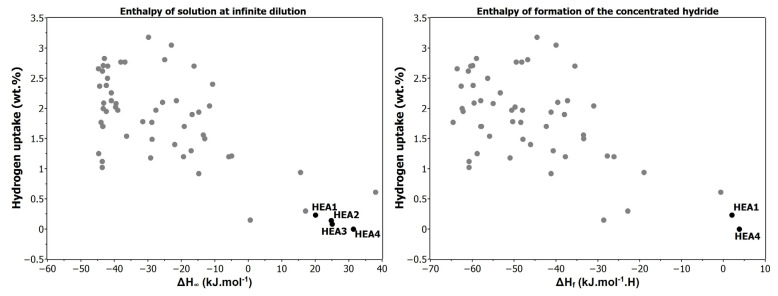
Left: the maximum hydrogen absorption as a function of the enthalpy of solution at infinite dilution (Δ*H_∞_*). Right: the maximum hydrogen absorption as a function of the enthalpy of formation of the concentrated hydride (Δ*H_f_*). Grey data points correspond to 55 alloys from the reference [16], while black data points represent our studied alloys.

We intentionally selected alloy compositions to maximize Δ*H_∞_* and Δ*H_f_*, aiming to create hydrogen-resistant materials. According to the theory, these positive enthalpy values should prevent hydride formation (Table 1, Figure 2).

### 2.2. Material Preparation

The equimolar CoNiMnCrFe (**HEA1**), CoNiMnCrCu (**HEA2**), CoNiMnFeCu (**HEA3**), and CoNiAlCrFe (**HEA4**) alloys were prepared from highly pure elements (>99.9%) by arc melting in the Mini Arc Melting System MAM-1 furnace (Edmund Bühler GmbH, Bodelshausen, Germany) under argon atmosphere. The amount of each alloy melted was approximately 3 g per sample. To ensure the homogeneity, the alloys were turned over and re-melted five times. The Ti getter pieces were melted before synthesizing the alloys to maintain a clean atmosphere in the melting chamber.

### 2.3. Material Characterization

The initial step involved determining the density of the alloys using the Archimedes method with precise laboratory scales, the Kern ABT 120-4M fitted with the specialized ABT-A01 adapter for density determination (KERN & SOHN GmbH, Balingen, Germany). A hardness test was made on the samples using a Wilson–Wolpert Tukon 1102 Vickers indenter, applying a load of 0.3 kg HV_0.3_ (Berg Engineering & Sales Company, Inc., Rolling Meadows, IL, USA). Ten indentations were performed, and the average value along with the standard deviation was calculated from these measurements. Metallographic cuts were made from the bulk alloys by the standard method, including casting in resin, followed by grinding and polishing. Chemical composition was determined using energy-dispersive X-ray spectroscopy (EDS) on a Jeol JSM 7000F scanning electron microscope (JEOL Ltd., Akishima, Tokyo, Japan). Samples were subsequently ground in a high-energy vibratory mill for 20 min under argon and sieved to a particle size below 45 μm. X-ray diffraction (XRD) patterns were collected on a Philips X’Pert Pro diffractometer (Malvern Panalytical, Malvern, The Netherlands) using Cu Kα radiation (1.5406 Å) in the 2θ range of 20° to 100° with a step size of 0.03° and a dwell time of 60 s per step.

### 2.4. Hydrogen Absorption Experiments

Measurements of hydrogen absorption were carried out using the magnetic suspension balance (TA Instruments, New Castle, DE, USA) that operates at pressures up to 50 MPa with an accuracy of 0.05%. The experiments were conducted on all the samples according to the following protocol:

1. Approximately 1 g of the powder alloy was placed in the reaction chamber of the magnetic suspension balance. The system was sealed and evacuated to a vacuum. The first hydrogen-absorption measurement began by filling the reaction chamber with hydrogen to a pressure of 2 MPa, followed by increasing the temperature from RT to 250 °C in 25 °C increments, with each step being held for 30 min. The sample mass was monitored by the magnetic suspension balance. Following this measurement, the sample was cooled to room temperature under a hydrogen pressure of 2 MPa.
2. Next, the chamber was evacuated and heated to 400 °C for 3 h to desorb hydrogen from the sample and then cooled to RT.
3. The reaction chamber was heated to 250 °C. After reaching the required temperature, the system was filled with hydrogen to a pressure of 2 MPa. The measurement was held for 30 min.

The raw hydrogen absorption data were recalculated to reflect the amount of hydrogen absorbed per gram of material, applying standard routines for buoyancy corrections [22].

## 3. Results and Discussion

**Figure 3:**
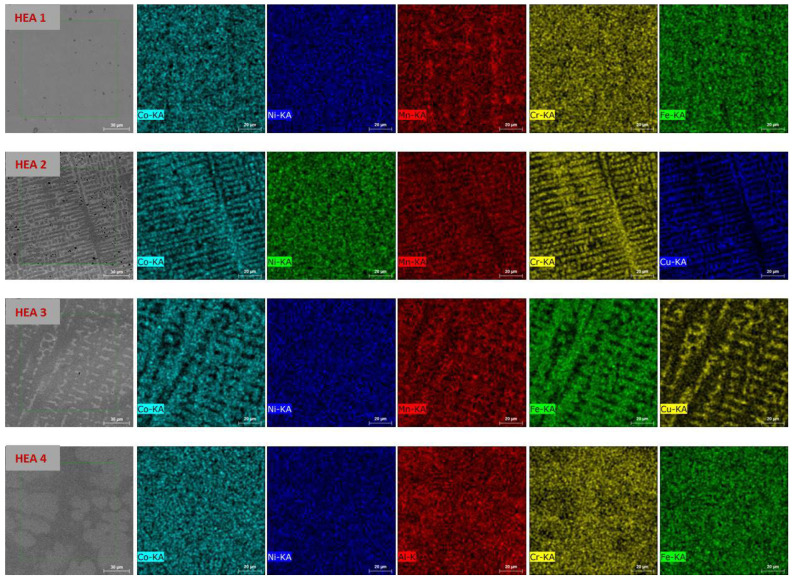
Microstructure and EDX elemental maps of all four alloys (**HEA1**, **HEA2**, **HEA3**, and **HEA4**). The grayscale image on the left represents the microstructure, while the colored maps correspond to the distribution of individual elements (Co, Ni, Mn, Cr, Fe, Cu, and Al) across the surface. These maps confirm the homogeneity of the alloys and the uniform distribution of elements within the investigated regions.

Four alloys (CoNiMnCrFe, CoNiMnCrCu, CoNiMnFeCu, and CoNiAlCrFe), all predicted to form single-phase highly supersaturated solid solutions, were prepared. The microstructures of the prepared high-entropy alloys (HEAs) were analyzed using a scanning electron microscope equipped with energy-dispersive X-ray spectroscopy (EDS), with results visualized in Figure 3. The microstructures of the alloys exhibit notable differences, with dendritic patterns visible in certain compositions, such as **HEA2** and **HEA3**. These dendritic features are indicative of compositional segregation during solidification, which is common in multicomponent alloys. In contrast, the microstructures of **HEA1** and **HEA4** appeared more homogeneous, reflecting differences in elemental interactions and solidification behavior. The EDX elemental mapping confirms the uniform distribution of alloying elements within each alloy. For **HEA1**, the Co, Ni, Mn, Cr, and Fe elements were evenly distributed, consistent with its equimolar composition. Similarly, the mapping for **HEA2** and **HEA3** showed a uniform spread of Co, Ni, Mn, Cr, Cu, and Fe, despite the visibly dendritic structures. In **HEA4**, the substitution of Al for Mn resulted in a homogeneous microstructure.

The densities of the alloys was similar, ranging from 7.6 to 7.99 g∙cm^−^³, reflecting the close atomic packing and comparable elemental compositions of the alloys. However, their hardnesses (Table 2) varied significantly. FCC alloys had a hardness of 130–220 HV_0.3_, while the BCC alloy was much harder, with a value of around 500 HV_0.3_.

**Figure 4:**
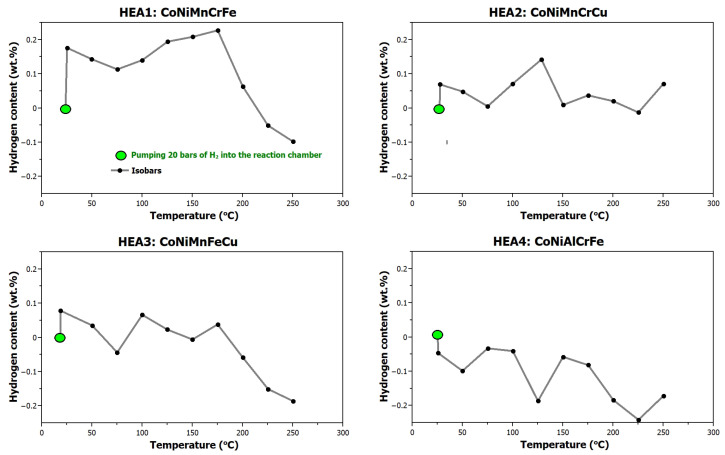
Hydrogen absorption of the studied alloys (isobars).

The alloys were subjected to hydrogen charging at 2 MPa pressure within a temperature range of RT to 250 °C, see Figure 4. Experimental results indicate negligible hydrogen absorption, with a maximum uptake of 0.23 wt.%, H/M = 0.13 (**HEA1**) at a temperature of 175 °C, confirming resistance to interstitial hydride formation. At elevated temperatures, hydrogen desorption becomes thermodynamically favorable because of the endothermic nature of hydrogen desorption and the increase in thermal energy. Specifically, as the temperature increases, the solubility of hydrogen in the alloy decreases, leading to a reduction in absorbed hydrogen. Moreover, higher temperatures provide sufficient energy for hydrogen atoms to overcome binding energies within the alloy, promoting desorption.

**Figure 5:**
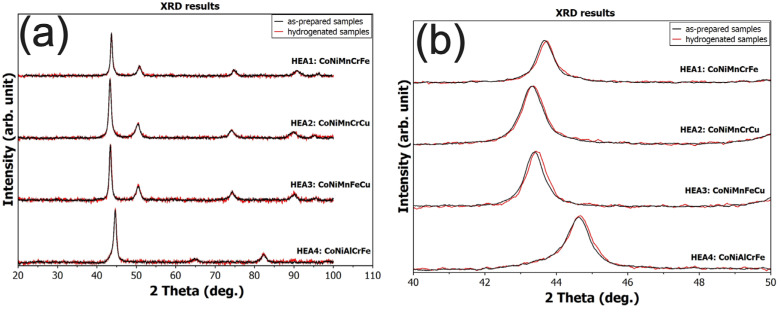
(**a**) The XRD patterns of the prepared samples (black curves) compared to the hydrogenated samples (red curves), showing no observable structural changes after hydrogen exposure across all tested alloys. (**b**) A magnified view of the largest peaks, around 45 degrees for each alloy, further illustrating the absence of any significant shifts or changes in peak intensity and confirming the structural stability of the alloys during hydrogenation.

To verify whether phase changes occurred in the alloys during hydrogen exposure, XRD measurements were performed both before and after hydrogenation. In Figure 5, the XRD patterns before hydrogenation are shown in black, while those after the hydrogen experiments are shown in red. This comparison demonstrates that the overlapping patterns prove the alloys remained unaffected by hydrogen in all cases. Rietveld refinement of the structural parameters showed a change in lattice parameters of only <0.002, which is at the level of accuracy of standard diffractometers.

## 4. Conclusions

This study presents a novel approach to alloy design by experimentally validating the semi-empirical model of Griessen and Driessen, targeting the development of high-entropy alloys (HEAs) that exhibit minimal hydrogen absorption. Unlike conventional research, which focuses on maximizing hydrogen storage capacity, our work uniquely demonstrates the potential to design alloys resistant to hydrogen uptake, addressing critical challenges in hydrogen-related applications where embrittlement and structural degradation are concerns. Four equiatomic HEAs were synthesized and systematically evaluated under constant hydrogen pressure (2 MPa) and a temperature range up to 250 °C. The results reveal that the alloys absorbed negligible amounts of hydrogen, with a maximum uptake of 0.23 wt.% for HEA1. Furthermore, at elevated temperatures, some alloys (e.g., HEA1 and HEA3) exhibited desorption of hydrogen, further confirming their resistance to long-term hydrogen retention. XRD analysis before and after hydrogenation showed no structural changes, highlighting the alloys’ remarkable stability under hydrogen exposure. This study also demonstrated that thermodynamic parameters, such as highly endothermic enthalpies of formation, play a critical role in achieving hydrogen-resistant behaviour. It is worth noting that the enthalpy values of several important metals and metalloids, such as Zn, Cu, Ca, Ag, Sn, W, and Si, have not yet been determined. This knowledge gap should merit the attention of experts in theoretical physics and chemistry in the current era of hydrogen technologies.

## Figures and Tables

**Table 1:** Thermodynamic parameters of the investigated alloys.

Alloy	*δ* [%]	Δ*H_mix_* [kJ∙mol^−1^]	Δ*H_∞_* [kJ∙mol^−1^]	Δ*H_f_* [kJ∙mol^−1^]	*Ω*	Δ*S_mix_* [J∙K^−1^∙mol^−1^]	*T_m_* [K]	*VEC*
**HEA1**: CoNiMnCrFe	3.01	−4.08	20.08	2.05	5.90	1.60 *R*	1808	8.01
**HEA2**: CoNiMnCrCu	2.98	+0.94	24.79	-	24.21	1.61 *R*	1701	8.66
**HEA3**: CoNiMnFeCu	3.02	+2.02	25.03	-	10.85	1.61 *R*	1643	9.06
**HEA4**: CoNiAlCrFe	5.78	−12.32	31.40	3.8	1.82	1.61 *R*	1673	7.20

**Table 2:** Results of the measurements of the studied alloys.

Alloy EDX Composition [at.%]	Density [g∙cm^−3^]	Hardness HV_0.3_	XRD	Temperature of max. H_2_ Absorption [°C]	Maximum H_2_ Capacity [wt.%] (H/M)
**HEA1** CoNiMnCrFe Co_21_Ni_20_Mn_16_Cr_22_Fe_21_	7.99	130 ± 5	FCC	175	0.23 (0.13)
**HEA2** CoNiMnCrCu Co_21_Ni_20_Mn_18_Cr_20_Cu_21_	7.81	220 ± 4	FCC	125	0.14 (0.08)
**HEA3** CoNiMnFeCu Co_21_Ni_21_Mn_17_Fe_21_Cu_20_	7.99	170 ± 5	FCC	RT	0.08 (0.04)
**HEA4** CoNiAlCrFe Co_20_Ni_20_Al_20_Cr_20_Fe_20_	7.06	492 ± 11	BCC	RT	0 (0)

## Data Availability

Data are contained within the article.
